# Prevalence of canine obesity in the city of São Paulo, Brazil

**DOI:** 10.1038/s41598-020-70937-8

**Published:** 2020-08-21

**Authors:** Mariana Yukari Hayasaki Porsani, Fabio Alves Teixeira, Vinicius Vasques Oliveira, Vivian Pedrinelli, Ricardo Augusto Dias, Alexander James German, Marcio Antonio Brunetto

**Affiliations:** 1 School of Veterinary Medicine and Animal Science, University of Sao Paulo (USP) – Department of Animal Nutrition and Production, Sao Paulo, Postcode 05508-010 Brazil; 2 Institute of Ageing and Chronic Disease, University of Liverpool, Liverpool, Postcode L7 8TX UK

**Keywords:** Nutrition, Obesity

## Abstract

Canine obesity is associated with comorbidities, a shortened lifespan, and a poorer quality of life, but epidemiological studies characterizing canine obesity in Latin America are scarce. Therefore, this study aimed to determine the prevalence of canine obesity in the city of Sao Paulo, Brazil, and the possible associated causal factors. Randomly-selected households from different city regions were visited. Dogs in each household were evaluated and owners completed a questionnaire whilst their anthropometric measures were taken. Total of 285 dogs from 221 owners were included, and the combined prevalence of overweight and obesity was 40.5%. The prevalence of overweight and obesity was greater in female dogs (*P* = 0.003) and in dogs that were neutered (*P* = 0.001). There was also a positive association between BCS and frequency of visits to a veterinarian (*P* = 0.026), feeding frequency (*P* = 0.033), and higher snack intake (*P* = 0.011). Further, the BCS of dogs was greater when their owners reported consuming more snacks themselves (P = 0.005) and whose had a presence of elderly people in the household (P = 0.006). In conclusion, the prevalence of obesity found in a Brazilian metropolitan region was similar to that if other countries, and neutering and snack intake were associated with the development of this disease.

## Introduction

Obesity is characterized by the accumulation of adipose tissue to the point that health is adversely affected, (BROOKS)^1^ and adverse effects in dogs include comorbidities^2^, reduced quality of life, and a shortened lifespan^3^ (Alonso). Body condition scoring (BCS) is the current method used to determining adiposity, with a 9-unit system most widely recommended^4^. Using such a system, dogs with a BCS of 6 or 7 are defined as having an overweight condition, whilst those with a BCS of 8 or 9 have obesity. There has been a significant increase in obesity prevalence over the last 30 years^5^, with the current prevalence of obesity. The estimate obese and overweight dog population assessed in veterinary hospitals has been determined in the United States, United Kingdom, China, Japan, and Spain, which place obesity prevalence at 5 to 20% and overweight between 20 and 30%^2,6–8^. Several factors are associated with the development of obesity including genetics, environmental, behavioral, and sociocultural factors^6,9–11^. Owner factors are also implicated, including feeding practices (e.g. offering excessive food) and providing physical activity. Sometimes, owner behavior results from misinformation about appropriate pet care, and they might even transfer their unhealthy feeding habits to their pets^6,7,11^.

Until now, information has been scarce regarding canine obesity epidemiology and owner profile in Latin America. Most notably, the current prevalence of overweight and obesity in the canine pet population is not known, whilst more information is required regarding owner factors associated with its development in this region. Therefore, this study aimed to estimate the current prevalence of overweight and obesity in pet dogs in the city of Sao Paulo, Brazil, and the factors associated with this disease.

## Material and methods

### Study design and ethical considerations

This was a cross-sectional study to determine prevalence and risk factors associated with obesity in Brazil. It was conducted in the city of Sao Paulo between November 2017 and November 2018. The study employed cluster sampling, with dogs defined as the sample unit. The experimental protocol was conducted according to ethical principles in human and animal experimentation and was approved by the Commission on Ethics in the Use of Animals of the School of Veterinary Medicine and Animal Science of the University of Sao Paulo (protocol number 3443010217) and the Commission of Ethics in Research with Humans of the Luiz de Queiroz College of Agriculture of the University of Sao Paulo (protocol number 71711317.2.0000.5395). Before participating, owners were fully a verbal explanation informed about all aspects of the study, and gave their consent in writing.

### Household visits, estimated sample size and sampling strategy

Household visits were conducted in the city of Sao Paulo by two study investigators (MYHP and FAT) together. The minimum sample size of dogs to be evaluated was 196, estimated by the equation of prevalence, with a significance level of 95% and an error of 5%^12^. In a previous study that determined the number of pet dogs in the city of Sao Paulo^14^, 50% of households contained a mean of two dogs. Therefore, it was determined that at least 200 households would need to be visited in order to achieve the appropriate sample size. Twenty of the 18,228 urban census areas of Sao Paulo^13^ were randomly selected and visited. These areas were plotted on a map using Google Earth (https://earth.google.com/web/), and the Google Maps app (iPhone version 11.1.2, Apple, United States) was used to determine the route to follow for each region, ensuring that at least ten households per selected area could be visited.

The sequence of households visited was initially determined by randomly drawing the first household with the software Office 15 Microsoft Excel (2013), based on the number of households of that specific census area^13^.

For the definition of the first house to be visited a random draw was performed with Excel 2013 (Microsoft, United States) based on the number of households in the censitary region according to IBGE^13^. Equation (1) was used to determine the interval between households to be visited.

1 $$ X = \left( \frac{Y}{10} \right) $$

X = Interval between households to be visited; Y = Total number of households in the region; 10 = Minimum number of houses to be visited in each region.

If data collection from a particular household was unsuccessful, the house immediately next to the selected one was instead visited, and so on, until data were successfully gathered. The reasons for unsuccessful data collection were categorized as follows: the owner that did not know all the required information about their dog; the dog was aggressive (meaning that a BCS assessment could not be performed) animals; the dog was less than eight months of age; there were no dogs in the households; the household owner was not present at the property; either the dog or the owner was pregnant; or the household owners refused to participate in the study.

The visits in all regions were made during business hours throughout a year. In the households that had more than one dog, all were evaluated. Only one owner was evaluated in each household visited, which was the person in charge of handling the dogs.

### Information gathering

**Figure 1:**
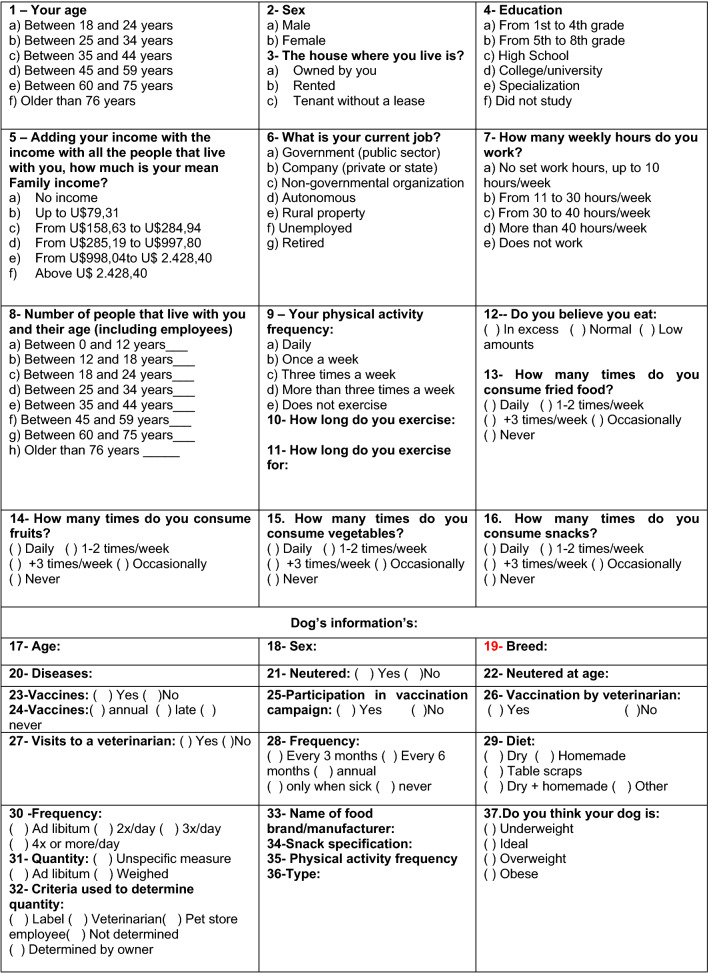
Details of the questionnaire completed by participating owners.

Owners were asked to answer a questionnaire (Fig. 1) regarding the health, feeding behavior and general management of their dog, as well as owner’s feeding habits and socioeconomic condition. During visits, the BCS of the dog was assessed, the body mass index (BMI) of the owner was measured, and other morphometric measurements were taken from the owner. Questionnaires were administered by the same two veterinarians (MYHP and FAT) in the same time, and owners answered questions about their socioeconomic status, feeding habits, and exercise habits. Feeding habits were classified according to the Feeding Guide of the Brazilian Population^14^, with individuals consuming snacks three or more times a week or consuming fruits and vegetables once or twice a week (or less) were considered to have ‘unhealthy’ feeding habits. The owner´s income was asked in reais (currency of Brazil), and then converted to American Dollars for the purpose of data analysis (https://www.conversor-dolar.com.br, access in October 2019). Owners' income was classified according to Neri^15^ in which households were considered to have a low income if annual income was U$3,419,28; middle income if between U$ 3,422,28 and U$11,976,48; and elite if income was above U$11,976,48. The BCS assessments were performed by two trained veterinarians (MYHP and FAT), according to a 9-point scale^4^, whilst age range of dogs was classified according to breed size: small breeds (0–10 kg) as young (< 7 years), middle-aged (7 to 12 years) and senior (> 12 years); medium breeds as young (< 6 years), middle-aged (6 to 10 years) and senior (> 10 years); large breeds as young (< 5 years), middle-aged (5 to 9 years) and senior (> 9 years); and giant breeds as young (< 3 years), middle-aged (3 to 7 years) and senior (> 7 years)^16^.

Owners had their BMI evaluated according to the methodology recommended by the World Health Organization^17^. Height was measured using metric tape, whilst weight was measured using portable digital scales (Supermedy, Barueri, Sao Paulo, Brazil) which were regularly calibrated for precision and accuracy using test weights (Oxer Ltd.). BMIs were classified as underweight if values were below 18.5; eutrophic if values were between 18.6 and 24.9; overweight if values were between 25.0 and 29.9; and obese if values were ≥ 30.0. Morphometric measurements were made by metric tape according to the methodology described by the World Health Organization^17,18^ and included the abdominal and hip circumference. Standard cut-points were applied: a waist/height ratio of > 0.52 was considered to be a potential health risk, whilst waist/hip ratios of < 0.91 for men and < 0.76 for women were considered to be a low cardiovascular disease risk^19^.

The frequency of physical activity was classified using the system described by Degeling et al.^20^ With this system, a total weekly activity of ≤ 150 and > 150 min were classed as low and high weekly activity, respectively; further, ≤ 30 min per day was considered to be low daily activity; between 30 and 120 min per day was considered to be moderate daily activity, and > 120 min per day was considered to be high daily activity. The owner activity was classified based on the answer of the questionnaire.

### Data handling and statistical analysis

To estimate the prevalence of dogs with overweight and obesity in the city of Sao Paulo, two mathematical weightings were considered for each animal, one related to the impact of the animal in its census area and another related to the impact of the census area in all city regions. The weightings were determined using a two-stage method and the weighting of each household and each animal in the household were determined according to Canatto et al.^21^.

Questionnaire responses were initially evaluated as percentages. The chi-square test, with a significance level of 5%, was used to assess the differences between BCS [underweight (BCS ≤ 3); ideal (BCS 4 and 5); overweight (BCS 6 and 7); and obese (BCS 8 and 9)] and different variables including reported diseases, feeding management of dogs, and owner characteristics. Dog owner variables assessed included age (with dogs classified as young, adult and senior), sex, reproductive status (whether neutered or intact and age at neutering), and breed size. Although details of individual breeds were reported, these were not assessed statistically given small studies. For a similar reason, details of diseases reported by the owner were not assessed statistically. Variables analyzed with this test were later evaluated with the multiple correspondence test to investigate profiles regarding BCS of dogs. This analysis was performed with computer software (“R” Studio with the package “ca”, version 1.2.5032, R Studio, United States).

Risk factors for overweight and obese body condition were determined by calculating odds ratios (OR) and associated 95% confidence intervals (CI 95), using multiple logistic regression. For this analysis, dogs with BCS ≥ 4 were divided into two categories: overweight and obese (BCS > 5) vs. ideal weight (BCS 4 and 5). Initially, variables were tested independently using the Chi-square test, and those that were *P* < 0.20 were used in multiple regression. The model was refined in a backwards stepwise fashion, by removal of the least significant variable at each round until the model only contained variables that were *P* < 0.05. These analyses were performed with computer software (SPSS, version 20; IBM Corporation).

Kappa analysis was used to determine agreement between BCS determined by the veterinarians and owners, with results being interpreted according to Landis and According (1977)^22^, whereby agreement is assumed to be low if between 0.00 and 0.20; reasonable if between 0.21 and 0.40; moderate if between 0.41 and 0.60; high if between 0.61 and 0.80; and almost perfect if between 0.81 and 1.00, all performed with computer software (SPSS, version 20; IBM Corporation).

### Ethical approval

The experimental protocol was conducted according to ethical principles in human and animal experimentation and was approved by the Commission on Ethics in the Use of Animals of the School of Veterinary Medicine and Animal Science of the University of Sao Paulo (protocol number 3443010217) and the Commission of Ethics in Research with Humans of the Luiz de Queiroz College of Agriculture of the University of Sao Paulo (protocol number 71711317.2.0000.5395).

## Results

### Characteristics of the final study population

A total of 1,198 households were visited and of these, 221 were included in the study comprising a total of 285 dogs. Reasons for exclusion included: households with no house owner present (619), households without dogs (250), owner declining participation in the study (50), residential buildings with unauthorized access (25), homes with dogs but owners absent (18), aggressive dogs (8); pregnant dogs or owners (3); dogs less than 8 months old (2), schools with unauthorized access (2).

Based upon BCS determined by the investigators, 23 dogs (8.1%) were classified as underweight, 149 (52.3%) were classified as ideal weight, 75 (26.3%) were classified as overweight and 38 (13.3%) were classified as obese. After weighting to account for the city region, the overall prevalence in the city of Sao Paulo, Brazil, was estimated at 25.9% for dogs in overweight status and 14.6% for dogs with obesity.

### Associations between bodyweight and animal variables

Information regarding age range, sex, breed, breed size, reproductive status, age at neutering, and reported diseases are described in Tables 1 and 2. Body condition score was associated with sex (*P* = 0.003) and reproductive status (*P* < 0.001), with the prevalence of overweight and obesity being greater in female dogs and those that were neutered. However, body condition was not associated with age, breed size, or age of neutering.

**Table 1:** Relationship between signalment and the body condition of dogs.

Characteristics	Underweight (BCS 1–3)		Ideal (BCS 4–5)		Overweight (BCS 6–7)		Obese (BCS 8–9)		All dogs		P-value^a^
	N	%	N	%	N	%	N	%	N	%	
**Age range**^b^											
Young	12	7.1	98	58.0	40	23.7	19	11.2	169	100.0	0.191
Adult	4	6.1	30	45.5	21	31.8	11	16.7	66	100.0	
Senior	7	14.9	18	38.3	14	29.8	8	17.0	47	100.0	
No information	0	0.0	3	100.0	0	0.0	0	0.0	0	100.0	
**Sex**											
Female	11	7.3	65	43.3	46	30.7	28	18.7	150	100.0	0.003
Male	12	8.9	84	62.2	29	21.5	10	7.4	135	100.0	
**Reproductive status**											
Neutered	8	6.4	50	40.0	39	31.2	28	22.4	125	100.0	< 0.001
Intact	15	9.4	99	61.9	36	22.5	10	6.2	160	100.0	
**Sex and reproductive status**											
Neutered females	7	8.9	29	36.7	24	30.4	19	24.1	79	100.0	0.404
Neutered males	1	2.2	21	45.7	15	32.6	9	19.6	46	100.0	
**Age at neutering**											
Up to 1 year	4	5.3	36	47.4	21	27.6	15	19.5	76	100.0	0.359
1 to 3 years	1	10.0	2	20.0	4	40.0	3	30.0	10	100.0	
More than 3 years	2	7.7	6	23.1	10	38.5	8	30.8	26	100.0	
Intact	15	9.4	99	61.9	36	22.5	10	6.2	160	100.0	
No information	1	7.7	6	46.1	4	30.8	2	15.4	13	100.0	

*BCS* body condition score.

^a^p value obtained by the chi-square test.

^b^Age and breed size classification according to Hosgood and Scholl^16^.

**Table 2:** Relationship between signalment and health information and the body condition of dogs.

Characteristics	Underweight (BCS 1–3)		Ideal (BCS 4–5)		Overweight (BCS 6–7)		Obese (BCS 8–9)		All dogs		P-value^a^
	N	%	N	%	N	%	N	%	N	%	
**Breed**											
Dobermann Pinscher	1	5.6	8	44.4	5	27.8	4	22.2	18	100.0	-
Labrador Retriever	1	10.0	2	20.0	4	40.0	3	30.0	10	100.0	
Lhasa Apso	2	20.0	8	80.0	0	0.0	0	0.0	10	100.0	
Poodle	3	18.8	5	31.2	5	31.2	3	18.8	16	100.0	
Shih-tzu	0	0.0	13	81.2	3	18.8	0	0.0	16	100.0	
Mixed breed	12	8.7	74	53.6	37	26.8	15	10.9	138	100.0	
Yorkshire Terrier	1	4.3	11	47.8	5	21.7	6	26.1	23	100.0	
Other breeds	3	5.6	28	51.9	16	29.6	7	13.0	54	100.0	
**Breed size**^b^											
Small	16	10.3	78	50.0	41	26.3	21	13.5	156	100.0	0.637
Medium	5	6.0	50	59.5	21	25	8	9.5	84	100.0	
Large	2	4.4	21	46.7	13	28.9	9	20.0	45	100.0	
**Disease reported by the owner**											
Cardiopathy	1	100.0	0	0.0	0	0.0	0	0.0	1	100.0	-
Tracheal collapse	0	0.0	0	0.0	0	0.0	1	100.0	1	100.0	
Dermatopathy	0	0.0	3	60.0	1	20.0	1	20	5	100.0	
Diabetes mellitus	0	0.0	2	66.7	1	33.3	0	0.0	3	100.0	
Epilepsy	0	0.0	2	100.0	0	0.0	0	0.0	2	100.0	
Neoplasia	0	0.0	1	50.0	1	50.0	0	0.0	2	100.0	
Orthopedic	0	0.0	5	62.5	1	12.5	2	25	8	100.0	
No disease	22	9.6	136	59.4	71	31.0	0	0.0	229	100.0	

*BCS* body condition score.

^a^p value obtained by the chi-square test.

^b^Breed size classification according to Hosgood and Scholl^16^

Information regarding veterinary care (e.g. vaccination history and details of veterinary visits), exercise, other animals in the household, and type of household) and feeding management (type of diet, meal frequency and portion, snack intake) are described in Tables 3, 4 and 5. There were no associations between body condition and vaccination status (whether vaccinated, *P* = 0.863; frequency of vaccination, *P* = 0.178; whether vaccinated in a municipal campaign, *P* = 0.118; and vaccination by a veterinarian, *P* = 0.155) and whether the dog had been assessed by a veterinarian (*P* = 0.091). However, there was a significant association between body condition and the frequency of visits to a veterinarian (*P* = 0.026), with the prevalence of obesity being less in dogs that never visited a veterinarian.

**Table 3:** Association between body condition and the health information of the 285 evaluated dogs.

Characteristic	Underweight (BCS 1–3)		Ideal (BCS 4–5)		Overweight (BCS 6–7)		Obese (BCS 8–9)		All dogs		*P*-value^a^
	N	%	N	%	N	%	N	%	N	%	
**Vaccinated**											
Yes	22	8.0	143	52.0	73	26.5	37	13.5	275	100.0	0.863
No	1	16.7	4	66.7	1	16.7	0	0.0	6	100.0	
No information	0	0.0	2	50.0	1	25.0	1	25.0	4	100.0	
**Frequency of vaccination**											
Yearly	19	7.7	130	52.6	64	25.9	34	13.8	247	100.0	0.178
Not every year	3	10.7	13	46.4	9	32.14	3	10.7	28	100.0	
Never	1	16.7	4	66.7	1	16.7	0	0	6	100.0	
No information	0	0	2	50.0	1	25.0	1	25.0	4	100.0	
**Vaccinated in a municipal campaign**											
Yes	14	10.2	61	44.5	36	26.3	26	18.9	137	100.0	0.118
No	8	5.6	85	60.2	38	26.9	10	7.1	141	100.0	
No information	1	14.3	3	42.9	1	14.3	2	83.3	7	100.0	
**Vaccinated by veterinarian**											
Yes	15	6.9	114	52.3	55	25.2	34	15.6	218	100.0	0.155
No	8	11.9	35	52.2	20	29.9	4	6.0	67	100.0	
**Has been assessed by a veterinarian**											
Yes	17	7.0	125	51.2	65	26.6	37	15.2	244	100.0	0.091
No	6	15.8	23	60.5	10	23.7	0	0.0	39	100.0	
**Frequency of visits to a veterinarian**											
Yearly	2	2.3	54	61.4	19	21.6	13	14.8	88	100.0	0.026
Only when sick	16	10.1	73	45.9	46	28.9	24	15.1	159	100.0	
Never	4	12.9	19	61.3	8	25.8	0	0.0	31	100.0	
No information	1	14.3	3	42.9	2	28.6	1	14.3	7	100.0	

*BCS* body condition score.

^a^*P-*value obtained by the chi-square test.

**Table 4:** Association between body condition and general management of the 285 evaluated dogs.

Characteristic	Underweight (BCS 1–3)		Ideal BCS 4–5)		Overweight (BCS 6–7)		Obese (BCS 8–9)		All dogs		P-value^a^
	N	%	N	%	N	%	N	%	N	%	
**Type of diet**											
Homemade	1	8.3	7	58.3	2	16.7	2	16.7	12	100.0	0.864
Commercial	17	8.4	104	51.5	54	26.7	27	13.4	202	100.0	
Commercial, homemade, scraps	5	7.2	38	55.1	18	26.1	8	11.6	69	100.0	
No information	0	0.0	0	0.0	1	50.0	1	50.0	2	100.0	
**Meal frequency**											
Once a day	0	0.0	10	58.8	6	35.3	1	5.9	17	100.0	0.033
Twice a day	3	2.6	62	53.9	34	29.6	16	13.9	115	100.0	
≥ three times a day	5	10.2	31	63.3	9	18.4	4	8.2	49	100.0	
Ad libitum	15	14.7	46	45.1	25	24.5	16	15.7	102	100.0	
No information	0	0.0	0	0.0	1	50.0	1	50.0	2	100.0	
**Method of quantification of daily food intake**											
Not weighted	23	9.7	121	51.1	64	32.6	31	12.7	239	100.0	0.195
Weighed	0	0.0	28	60.9	11	23.9	7	15.2	46	100.0	
**Criteria to determine daily food intake**											
Dog breeder or shop worker	0	0.0	1	50.0	1	50.0	0	0.0	2	100.0	0.503
Label	0	0.0	17	73.9	3	13.0	3	13.0	23	100.0	
Veterinarian	0	0.0	22	55.3	11	28.9	6	15.8	39	100.0	
Owner choice	23	13.7	109	65.5	60	35.7	29	17.3	221	100.0	
No information	9	11.8	38	50.0	21	27.7	8	10.5	76	100.0	
**Snacks**											
Yes	10	5.1	102	52.0	52	26.5	32	16.3	196	100.0	0.011
No	13	14.6	47	52.8	23	25.8	6	6.7	89	100.0	
**Type of snack**											
Human	3	3.9	38	50.0	17	22.4	18	23.7	76	100.0	0.271
Canine	1	2.6	22	57.9	9	23.7	6	15.8	38	100.0	
Human and canine	6	7.3	42	51.2	26	31.7	8	9.7	82	100.0	
Apartment	4	5.2	42	55.2	18	23.7	12	15.8	76	100.0	

*BCS* body condition score.

^a^*P-*value obtained by the chi-square test.

**Table 5:** Association between body condition and the physical activity of the 285 evaluated dogs.

Characteristic	Underweight (BCS 1–3)		Ideal (BCS 4–5)		Overweight (BCS 6–7)		Obese (BCS 8–9)		All dogs		P-value^a^
	N	%	N	%	N	%	N	%	N	%	
**Daily exercise**^b^											
Low	3	9.4	21	65.6	7	21.9	1	3.1	32	100.0	0.265
Moderate	0	0.0	15	65.6	5	21.7	3	13.0	23	100.0	
Not exercised daily	20	8.7	113	48.1	63	27.4	34	14.8	230	100.0	
**Weekly exercise**^b^											
≤ 150 h	5	5.4	54	58.1	21	22.6	13	14.0	93	100.0	0.856
> 150 h	3	7.1	24	57.1	11	26.2	4	9.5	42	100.0	
Does not exercise	15	10.0	71	47.3	43	28.6	21	14.0	150	100.0	
**Presence of other animals in the household**											
Dogs	8	6.5	54	43.9	40	32.5	21	17.1	93	100.0	0.289
Cats	0	0.0	5	71.4	2	28.6	0	0.0	7	100.0	
Dogs and cats	0	0.0	6	85.7	1	14.3	0	0.0	7	100.0	
No other animals	15	10.1	84	56.7	32	21.6	17	11.5	148	100.0	
**Type of household**											
House	19	9.1	107	51.2	57	27.3	26	12.5	209	100.0	0.587
Apartment	4	5.2	42	55.2	18	23.7	12	15.8	76	100.0	

*BCS* body condition score.

^a^*P-*value obtained by the chi-square test.

^b^Exercise levels were classified according to Degeling et al.^17^.

Both meal frequency (*P* = 0.033) and snack intake (*P* = 0.011) were significantly associated with body condition, with the prevalence of obesity being greatest in dogs fed twice daily or ad libitum and in those fed snacks. However, there was no association between body condition and type of food (*P* = 0.864), the type of snacks fed (*P* = 0.271), daily exercise (*P* = 0.265), weekly exercise (*P* = 0.856), the presence of other animals in the household (*P* = 0.289), and the type of household (*P* = 0.587).

### Associations between bodyweight and owner variables

Information regarding socioeconomic characteristics of owners (Table 6), feeding and exercising habits (Table 7), BMI and morphometric measurements (Table 8) and profile of people living within the household (Table 9) were also obtained. There were no significant associations between body condition and sex, age, education, family income, exercise, BMI, and owner morphometric measurements (Tables 8, 9). There were also no differences between body condition of dogs and owner reported consumption of various foods (Table 7) including fried food (*P* = 0.339), fruit (*P* = 0.200), vegetables (*P* = 0.659). However, the prevalence of obesity was positively associated by owners who reported consuming more snacks (*P* = 0.005). There was no association between body condition and either the occupation of the owner and the presence of children within the household; however, the prevalence of overweight dogs was less in households where elderly people lived, compared with households without elderly people (*P* = 0.006).

**Table 6:** Relationship between owner socioeconomic factors and the body condition of dogs.

Characteristics	Underweight (BCS 1–3)		Ideal (BCS 4–5)		Overweight (BCS 6–7)		Obese (BCS 8–9)		All dogs		P-value^a^
	N	%	N	%	N	%	N	%	N	%	
**Gender**											
Male	6	4.3	53	38.1	54	38.8	26	18.9	139	100.0	0.800
Female	17	11.6	96	65.7	21	14.4	12	8.2	146	100.0	
**Age range**											
18 to 34 years	9	10.2	50	56.8	14	15.9	15	17.0	88	100.0	0.094
35 to 59 years	12	9.0	68	50.7	42	31.3	12	9.0	134	100.0	
≥ 60 years	2	3.2	31	49.2	19	30.1	11	17.5	63	100.0	
**Education**											
Did not finish middle school	1	50.0	1	50.0	0	0.0	0	0.0	2	100.0	0.240
Middle school	8	14.3	23	41.1	17	30.3	8	14.3	56	100.0	
High school	9	7.4	65	53.2	32	26.2	16	13.1	122	100.0	
College	4	3.9	59	57.8	25	24.5	14	13.7	102	100.0	
No information	1	33.3	1	33.3	1	33.33	0	0.0	3	100.0	
**Family income**^b^											
Low	7	12.5	25	44.6	18	32.1	6	10.7	56	100.0	0.533
Middle class	8	6.6	66	54.5	28	23.1	19	15.7	121	100.0	
Elite	6	5.9	58	56.8	26	25.4	12	11.7	102	100.0	
No information	2	33.3	0	0.0	3	50.0	1	16.7	6	100.0	

*BCS* body condition score.

^a^p value obtained by the chi-square test.

^b^Family income classified according to Neri^23^.

**Table 7:** Relationship between owner feeding and exercise habits and the body condition of dogs.

Characteristics	Underweight (BCS 1–3)		Ideal (BCS 4–5)		Overweight (BCS 6–7)		Obese (BCS 8–9)		All dogs		P-value^a^
	N	%	N	%	N	%	N	%	N	%	
**Physical activity**											
Yes	8	7.1	68	60.2	31	27.4	6	5.3	113	100.0	0.406
No	15	9.6	81	51.6	44	28.0	17	10.8	157	100.0	
**Fried food consumption**^b^											
Healthy	11	7.6	79	54.5	40	27.6	15	10.3	145	100.0	0.339
Unhealthy	12	8.6	70	50.0	35	25.0	23	16.4	140	100.0	
**Fruit consumption**^b^											
Healthy	15	7.7	97	49.5	57	29.1	27	13.8	196	100.0	0.200
Unhealthy	8	9.0	52	58.4	18	20.2	11	12.4	89	100.0	
**Vegetable consumption**^b^											
Healthy	10	7.0	86	60.1	27	18.9	20	14.0	143	100.0	0.659
Unhealthy	13	9.2	63	44.4	48	33.8	18	12.7	142	100.0	
**Snack consumption**^b^											
Healthy	7	5.6	81	64.8	27	21.6	10	8.0	125	100.0	0.005
Unhealthy	16	10.0	68	42.5	48	30.0	28	17.5	160	100.0	

*BCS* body condition score.

^a^*P*-value obtained by the chi-square test.

^b^Feeding habits were classified according to the Feeding Guide of the Brazilian Population, with individuals consuming snacks three or more times a week or consuming fruits and vegetables once or twice a week (or less) were considered to have ‘unhealthy’ feeding habits.

**Table 8:** Relationship between owner body mass index and morphometric measurements and the body condition of dogs.

Characteristic	Underweight (BCS 1–3)		Ideal (BCS 4–5)		Overweight (BCS 6–7)		Obese (BCS 8–9)		All dogs		P-value^a^
	N	%	N	%	N	%	N	%	N	%	
**Owner body mass index**											
Underweight (< 18.5)	1	20.0	2	40.0	1	20.0	1	20.0	5	100.0	0.384
Eutrophic (18.5–24.9)	9	7.7	60	51.3	34	29.1	14	12.0	117	100.0	
Overweight (25.0- 29.9)	8	10.0	36	45.0	22	27.5	14	17.5	80	100.0	
Obese (≥ 30.0)	3	3.9	50	64.9	15	19.5	9	11.7	77	100.0	
No information	2	33.3	1	16.7	3	50.0	0	0.0	6	100.0	
**Owner waist/hip ratio**^b^											
Low risk	6	7.1	47	55.3	23	27.1	9	10.6	85	100.0	0.254
Moderate risk	9	11.5	45	57.7	16	20.5	8	10.3	78	100.0	
High risk	6	5.2	55	47.8	33	28.7	21	18.3	115	100.0	
No information	2	28.6	2	28.6	3	42.8	0	0.0	7	100.0	
**Owner waist/height ratio**^c^											
Not at risk	12	8.7	70	50.7	39	28.3	17	12.3	138	100.0	0.626
At risk	9	6.4	78	55.3	33	23.4	21	14.9	141	100.0	
No information	2	33.3	1	16.7	3	50.0	0	0.0	6	100.0	
**Owner abdominal circumference**^d^											
Not at risk	10	8.1	68	54.8	32	25.8	14	11.3	124	100.0	0.788
At risk	12	7.6	80	50.6	42	26.6	24	15.2	158	100.0	
No information	1	33.3	1	33.3	1	33.3	0	0.0	3	100.0	

*BCS* body condition score

^a^p value obtained by the chi-square test.

^b^Owner waist/hip ratio classified by health disease risk as low risk (< 0.91 men; < 0.76 women); moderate risk (0.90–0.96; 0.76–0.83) and high risk (> 0.97 men; > 0.82 women)^20,21^.

^c^Owner waist/height ratio classified by cardiovascular disease not at risk (< 0.52) and at risk (> 0.52)^20,21^.

^d^Owner abdominal circumference classified by health disease risk at risk (≥ 94 cm men; ≥ 80 cm women)^20,21^.

**Table 9:** Relationship between owner occupation and household information and the body condition of dogs.

Characteristics	Underweight (BCS 1–3)		Ideal (BCS 4–5)		Overweight (BCS 6–7)		Obese (BCS 8–9)		All dogs		P-value^a^
	N	%	N	%	N	%	N	%	N	%	
**Owner’s occupation**											
Outside of home	9	6.5	80	53.6	39	28.3	16	11.6	138	100.0	0.465
Home	14	10.2	69	47.4	36	26.3	22	16.1	137	100.0	
**Children in the household**											
Yes	9	10.1	49	55.1	22	24.7	9	10.1	89	100.0	0.749
No	14	7.1	100	51.0	53	27.0	29	14.8	196	100.0	
**Elderly people in the household**											
Yes	10	5.7	106	60.9	34	19.5	24	13.8	174	100.0	0.006
No	13	11.7	43	38.7	41	36.9	14	12.6	111	100.0	

*BCS* body condition score.

^a^P-value obtained by the chi-square test.

### Multiple logistic regression analysis to determine factors association with weight status in dogs

The results for the multiple logistic regression are presented in Tables 10, 11, 12 and 13. The odds of having an overweight or obese body condition was greater in adult dogs (OR 0.57, CI-95 0.34–0.94), female dogs (OR 2.45; CI-95 1.48–4.06), neutered dogs (independent of sex, OR 2.88; CI-95 1.74–4.78); dogs living with elderly people in the household (OR 1.69, CI-95 1.14–3.14); and having other pets in the households (OR 1.89, CI-95 1.03–2.76).

**Table 10:** Multiple logistic regression analysis of dog´s factors associated with overweight or obese body condition in dogs.

Variable	Overweight and obese (BCS ≥ 6)		Ideal (BCS 4–5)		Total		OR	CI-95	P-value^a^
	N	%	N	%	N	%			
**Age range**^b^									
Young	59	37.6	98	62.4	157	100.0	0.57	0.34–0.94	0.020
Adult	51	51.5	48	48.5	99	100.0			
**Sex**									
Female	74	53.2	65	46.8	139	100.0	2.45	1.48–4.06	< 0.001
Male	39	31.7	84	68.3	123	100.0			
**Reproductive status**									
Neutered	67	57.3	50	42.7	117	100.0	2.88	1.74–4.78	< 0.001
Intact	46	31.7	99	68.3	145	100.0			
**Age at neutering**									
≤ 3 years	43	53.1	38	46.9	81	100.0	0.38	0.14–1.05	0.050
> 3 years	18	75.0	6	25.0	24	100.0			
**Meal frequency**									
Up to three times a day	57	44.2	72	55.8	129	100.0	1.13	0.69–1.85	0.360
Ad libitum	54	41.2	77	58.8	131	100.0			
**Quantification of food amount**									
Not accurately measured	93	43.5	121	56.5	214	100.0	1.19	0.62–2.29	0.356
Weighed	18	39.1	28	60.9	46	100.0			
**Snack consumption by the dog**									
Yes	84	45.2	102	54.8	186	100.0	1.33	0.77–2.30	0.184
No	29	38.2	47	61.8	76	100.0			
**Exercise**									
Yes	8	27.6	21	72.4	29	100.0	0.71	0.22–2.33	0.398
No	8	34.8	15	65.2	23	100.0			

*BCS* body condition score; *OR* odds ratio; *CI-95* 95% confidence interval.

^a^*P*-value obtained by the chi-square test.

^b^Age and breed size classification according to Hosgood and Scholl^16^. BMI* body mass index.

**Table 11:** Multiple logistic regression analysis of owner´s factors associated with overweight or obese body condition in dogs.

Variable	Overweight and obese (BCS ≥ 6)		Ideal (BCS 4–5)		Total		OR	CI-95	P-value^a^
	N	%	N	%	N	%			
**Owner’s body mass index**									
> 25.0	60	41.1	86	58.9	146	100.0	0.86	0.53–1.42	0.328
≤ 25.0	50	44.6	62	55.4	112	100.0			
**Other pets in the household**									
Yes	64	49.6	65	50.4	129	100.0	1.69	1.03–2.76	0.025
No	49	36.8	84	63.2	133	100.0			
**Household type**									
Apartment	30	41.7	42	58.3	72	100.0	0.92	0.53–1.59	0.440
House	83	43.7	107	56.3	190	100.0			
**Presence of children in the household**									
Yes	31	44.9	38	55.1	69	100.0	1.01	0.58–1.76	0.546
No	81	44.8	100	55.2	181	100.0			
**Presence of elderly people in the household**									
Yes	58	53.7	50	46.3	108	100.0	1.89	1.14–3.14	0.014
No	54	38.0	88	62.0	142	100.0			

*BCS* body condition score; *OR* odds ratio; *CI-95* 95% confidence interval.

^a^*P*-value obtained by the chi-square test. BMI* body mass index.

**Table 12:** Multiple logistic regression analysis of factors associated with overweight or obese body condition in dogs.

Variable	Overweight and obese (BCS ≥ 6)		Ideal (BCS 4–5)		Total		OR	CI-95	*P*-value^a^
	N	%	N	%	N	%			
**Age range**^b^									
Young	59	37.6	98	62.4	157	100.0	0.57	0.34–0.94	0.020
Adult	51	51.5	48	48.5	99	100.0			
**Sex**									
Female	74	53.2	65	46.8	139	100.0	2.45	1.48–4.06	< 0.001
Male	39	31.7	84	68.3	123	100.0			
**Reproductive status**									
Neutered	67	57.3	50	42.7	117	100.0	2.88	1.74–4.78	< 0.001
Intact	46	31.7	99	68.3	145	100.0			
**Age at neutering**									
≤ 3 years	43	53.1	38	46.9	81	100.0	0.38	0.14–1.05	0.050
> 3 years	18	75.0	6	25.0	24	100.0			
**Meal frequency**									
Up to three times a day	57	44.2	72	55.8	129	100.0	1.13	0.69–1.85	0.360
Ad libitum	54	41.2	77	58.8	131	100.0			
**Quantification of food amount**									
Not accurately measured	93	43.5	121	56.5	214	100.0	1.19	0.62–2.29	0.356
Weighed	18	39.1	28	60.9	46	100.0			
**Snack consumption by the dog**									
Yes	84	45.2	102	54.8	186	100.0	1.33	0.77–2.30	0.184
No	29	38.2	47	61.8	76	100.0			

BCS: body condition score; OR: odds ratio; CI-95: 95% confidence interval.

^a^*P*-value obtained by the chi-square test.

^b^Age and breed size classification according to Hosgood and Scholl^16^. BMI* body mass index.

**Table 13:** Comparison of agreement between body condition scores determined by owner and veterinarians.

Owner BCS	Underweight (BCS 1–3)		Ideal (BCS 4–5)		Overweight (BCS 6–7)		Obese (BCS 8–9)		P-value^a^	KP^b^
	N	%	N	%	N	%	N	%		
Underweight	6	26.1	11	7.4	0	0.0	0	0.0	< 0.001	0.285
Ideal	16	69.6	127	85.2	47	62.7	10	26.3		
Overweight	1	4.3	10	6.7	28	37.3	20	52.6		
Obese	0	0.0	1	0.7	0	0.0	8	21.1		
Total	23	100.0	149	100.0	75	100.0	38	100.0		

*BCS* body condition score; *OR* odds ratio.

^a^p value obtained by the chi-square test.

^b^KP correspondent to kappa test (inter-rater agreement).

### Multiple correspondence analysis to determine factors association with weight status in dogs

**Figure 2:**
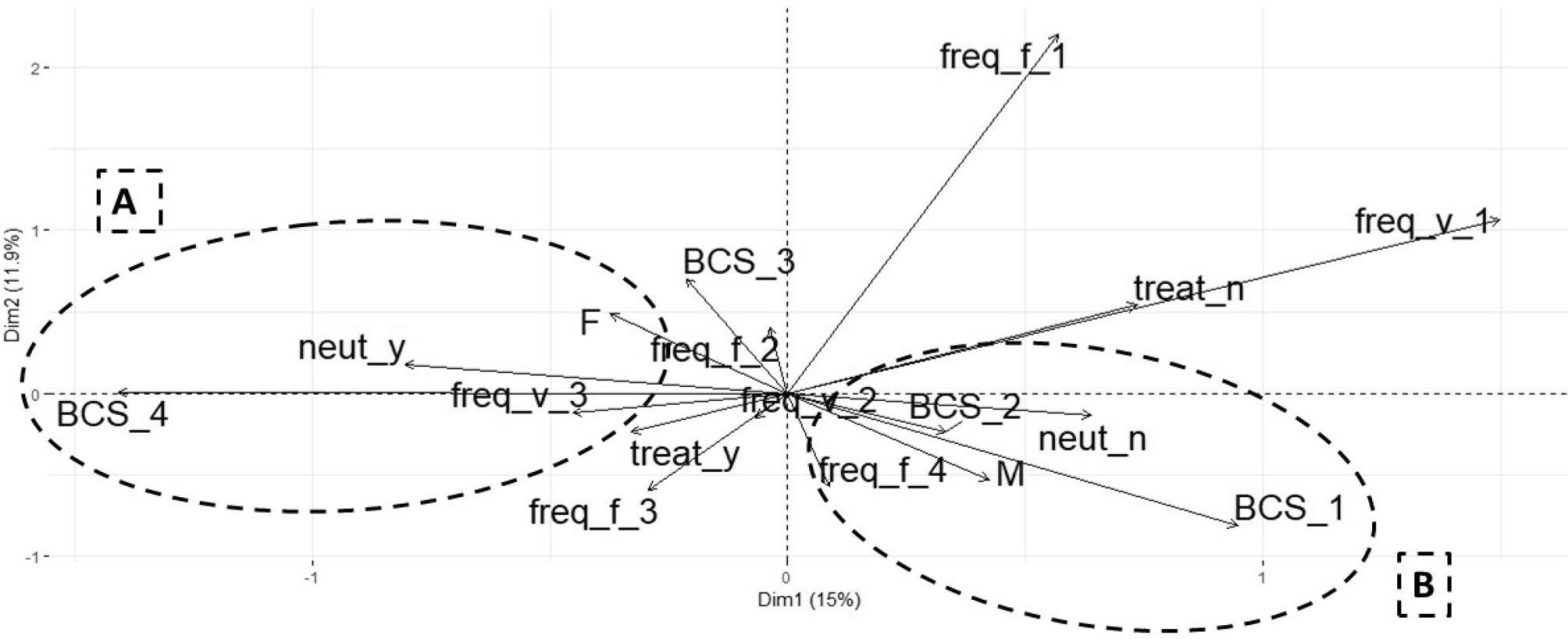
Multiple correspondence analysis of relationship between body condition score (BCS), underweight (BCS_1), ideal (BCS_2), overweight (BCS_3) and obese (BCS_4) with significant animal variables in the simple analysis: sex (sex_F = female and sex_M = male), reproductive status (neut_y = neutered and neut_n = non-neutered), daily frequency of feeding (freq._f_1 = 1 time, freq._f_2 = 2 times, freq._f_3 = 3 times and freq._f_4 = ad libitum), frequency of visits to veterinary practice (freq._v_1 = never, freq._v_2 = only if ill, freq._v_3 = frequently) and snacks intake (treat_y = yes, treat_n = no).

**Figure 3:**
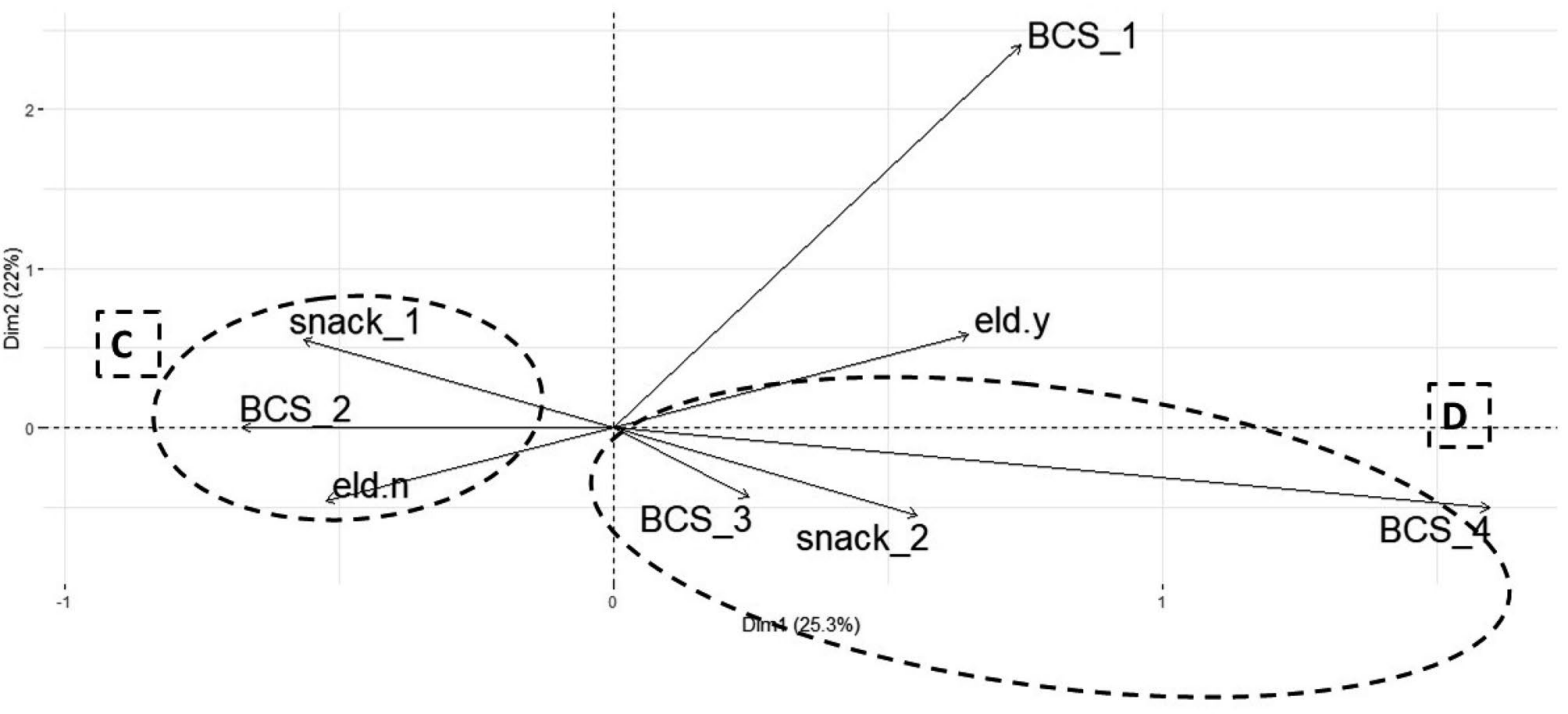
Multiple correspondence analysis of relationship between body condition score (BCS), for underweight (BCS_1), ideal (BCS_2), overweight (BCS_3) and obese (BCS_4) dogs, with significant animal variables in the simple analysis: presence of elderly people in the household (eld_y = house with elderly people and eld_n = house without elderly people) and habit of eating snacks (snack_1 = healthy habit and snack_2 = unhealthy habit).

The results of the multiple correspondence test are presented in Figs. 2 and 3. A stronger correspondence to obesity was seen for animals with profile A (neutered and with frequent visits to a veterinarian), whilst a stronger correspondence for ideal weight and underweight was seen for dogs with profile B (intact males fed ad libitum). Further, dogs with profile C (households without elderly people and owner with healthy feeding habits) had a stronger correspondence with ideal BCS, whilst dogs with profile D (household with elderly people and owner with unhealthy feeding habits) had a stronger correspondence with overweight and obesity.

Reasonable overall agreement (K = 0.285; *P* < 0.001), systematic differences were noted. For underweight dogs, more than 70% of owners overestimated the BCS of their dogs; for dogs in ideal BCS, 85% of the owners estimated their dog’s body condition correctly. Only 21% of owners of overweight and obese dogs accurately assessed their dog’s BCS, 63% underestimated the body condition, and 26% believed the dog to be in an ideal BCS.

## Discussion

To the authors’ knowledge, this is the first study to estimate the prevalence of canine obesity in Latin America, and the first to use a structured design akin to a census to estimate the obesity prevalence in a metropolitan area. Given that this approach evaluates a representative sample of a community, rather than animals registered with or assessed by veterinarians, it likely provides a more reliable estimate of true prevalence than studies with a population of veterinary hospitals^3,8,11,23–25^, retrospective BCS studies from hospital records^2,5,6,9,26,27^ or telephoning owners for information^28^. The prevalence of overweight condition was 25.9%, whilst the prevalence of obesity was 14.6%, results which are broadly similar to studies conducted in the United Kingdom, Japan, China, and Spain, where estimates of the combined prevalence of overweight and obesity ranged from 38 to 60%^2,3,5,8^.

In previous studies, several factors are reported to be associated with canine obesity including breed, sex, neutering, and owner habits including feeding and management practices of their dogs^8,29,30^. In this study, female sex was associated with overweight and obesity, with the odds being 2.45 times greater in female dogs compared with male dogs. The magnitude of this effect is similar to that obesity by Edney and Smith^11^_,_ and corroborating the findings of other studies^2,8,9,23,25^. Reasons previously suggested for such an association include a the fact that basal metabolic rate is less in female dogs, and the potential effects of estrogen on voluntary food intake ^28,29,31–33^. Reproductive status, independent of sex, was also associated with overweight and obesity, with the odds being 2.88 times greater for neutered dogs compared with those that were sexually intact. This finding is again consistent with the results of other studies, and is suggested to be related to altered behavior leading to food intake and decreased physical activity^25,28,33^. Although no association was found between age at neutering and body condition score, previous studies have suggested that early-age neutering can favor the maintenance of ideal body condition^33,34^. Age has also previously been associated with body condition in dogs, with the prevalence of overweight status being greater in middle age and senior dogs^2,5,6,9,23,27^. However, in the present study, there was no association between age and body condition. Previously-reported breed associations with obesity in dogs include Beagle, Dachshund, Golden Retriever, and Labrador Retriever^5,11,26^. In the current study, the greatest prevalence of overweight and obesity was seen in mixed-breed dogs, although 70% of Labrador Retrievers were overweight. The reasons in age and breed associations amongst studies are not clear, but might relate to differences in the demographics of the populations and also methods used in the study.

Feeding management practices of pet dogs are suggested to be associated with weight gain and obesity, including meal frequency, food choice, and how the portion size is determined^1^. Some epidemiological studies have suggested that feeding multiple meals a day decreases the risk of obesity^25,28^, possibly by increasing energy loss by thermogenesis^35^. However, the results of the current study did not support these findings, and the odds of overweight and obesity were greater in dogs fed three or more meals a day, a finding similar to other studies^2,11^. A strong correspondence was observed between ideal weight and underweight intact males and feeding ad libitum, as a result of a multiple correspondence analysis. However, this result is a controversy, because non-food restriction is considered an easy way to fed animals, and can contribute to consumption of excessive of calories (brooks)^1^. The results of the current study might reflect the fact that those feeding multiple meals also tended to feed a larger overall daily amount and the intact males may not have had an excessive motivation for food consumption. However, further studies would be required to confirm this possibility.

In addition, the type of food fed is also suggested to be important, not least given that consumption of commercial diets has previously been associated with a lower prevalence of obesity^2^. Establishing associations with particular food types proved to be difficult in the current study, because owners were often vague about exactly what they fed their dogs. Nonetheless, feeding snacks was associated with an increased odds of overweight or obesity in this study, consistent with findings from other studies^3,6,23,28,29,32^. Given that association between the of feeding snacks and canine obesity is such a consistent finding across many epidemiological studies, veterinarians should arguably provide clearer nutritional guidance to dog owners. Recommendations should be based on an appropriate nutritional assessment taking into consideration age, exercise level, breed, and other factors^1,29^. Indeed, a previous study from Germany suggested that owners frequently sort guidance from veterinarians about appropriate nutrition for their dogs^36^. Nonetheless, such an approach might be limited by the owners’ willingness to follow advice not least given that, in the current study most (65%) owners did not follow any recommendations of the type or quantity of food to feed their dog, either from the label or from a veterinarian.

Few studies evaluated the influence of feeding habits of owners on canine obesity, although some studies point to that relation^8,11^. In the present study, there was an association between ‘unhealthy’ feeding habits of owners and BCS of dogs, with 64% and 74% of owners of overweight and obese dogs, respectively, reporting such eating patterns. The reason for such an association is not known, but might reflect the fact that owners of dogs with overweight or obesity have less overall interest in ‘healthy nutrition’ than other owners. An alternative possibility is that, when owners snack on unhealthy foods, they also offer some to their dogs. Such a bystander effect might also explain the odds of obesity were 1.69 times greater in dogs that lived with other pets. Interestingly, there was no association between owners reporting ‘healthy feeding habits’ (such as consuming fruit and vegetables) and the body condition of their dogs. Thus, owners’ recognition of the need to consume fruits and vegetables regularly, might not offset the risks of weight gain from other feeding practices such as snacking. Nonetheless, these results should be interpreted cautiously because some owners might not have answered accurately, instead choosing to answer according to what they believed to be the correct so as to avoid judgment^11^.

Unlike previous epidemiological studies conducted in France, USA, and Spain^3,11,25^, in which the BMI of owners was associated with body condition of dogs, such an association was not observed in the current study, and nor were there any associations with any or the morphometric measurements. The reason for such a difference in not known, but might reflect the relatively high prevalence of overweight and obesity (55%) in the current study and also the fact that these were measured by the study investigators rather than self-reported. Research has shown that many people under-estimate their weight status and that of their children^37,38^. Similarly, owners often under-estimate the body condition of their dogs^11,39^, and such a finding was also seen in the current study. Nonetheless, owners of underweight dogs tended to over-estimate body condition, suggesting that owners tend to ‘normalize’, their dog’s body condition rather than systematically under-estimating it. Further work would be required to explore this finding more thoroughly.

Another factor that may contribute to increased BCS in dogs is the presence of older adults in the household^3,8,25^. In the current study, the odds of overweight or obesity were 1.89 times greater in households with of older people. Elderly people might be less spend more time with pets, which increases the likelihood of giving extra food or snacks between meals and, therefore, increasing energy intake^11,25,27^. Alternatively, elderly owners might be less able to exercise their dogs. Further studies would be needed to confirm the reasons for such findings.

The household income and education level of owners have been previously related to human feeding habits, where people of lower income and education level are more likely to eat unhealthily^40–43^. In one previous study, socioeconomic status of owners was associated with canine obesity^8^ but this was neither seen in the current study nor in another previous stud^3^. Although apartment dwelling has been associated with canine obesity in previous studies^2,25^, such an association was not observed in the present study. However, only the type of household was considered, and the actual living space was not taken into account; indeed, based on the authors’ observations, the living space in some apartments visited was greater than that in some of the houses visited. Future studies should consider not only type of household but the space available.

This study has some limitations, however households were approached on commercial hours only which may have influenced in the population due to inclusion of more retired or unemployed owners. Another limitation is that access to external areas of the household, when present, or size of household were not investigated, which could have influenced results regarding type of household as several apartments were bigger than houses. Regarding feeding habits informed by owners in the questionnaire, an involuntary bias could have happened due to defensive behavior of owners before some questions for fear of being judged and therefore answering what they think is correct rather than what reflects reality^44^.

## Conclusion

The prevalence of canine overweight and obesity observed in the present study was 40.5%. Factors associated with the development of obesity were sex, reproductive status, frequency of visits to a veterinarian, and feeding practices such as snack intake. The presence of elderly people and the owner’s unhealthy eating habits were also related to a higher body condition score and increased chances of gaining weight. This is the first Latin American epidemiology study regarding canine obesity, which is the most common nutritional disease in pets and is increasing in the last decade. Therefore, it is an important step into understanding factors that are correlated with the development of this disease and by doing so, understanding how to better approach owners in order to prevent and treat it.

## Data Availability

The datasets generated during and/or analyzed in the current study are available from the corresponding author on reasonable request.
